# Interactive effect of microstructure and cavity dimension on filling behavior in micro coining of pure nickel

**DOI:** 10.1038/srep23895

**Published:** 2016-04-06

**Authors:** Chuanjie Wang, Chunju Wang, Jie Xu, Peng Zhang, Debin Shan, Bin Guo

**Affiliations:** 1 School of Materials Science and Engineering, Harbin Institute of Technology, Harbin 150001, China; 2 Key Laboratory of Micro-Systems and Micro-Structures Manufacturing, Ministry of Education, Harbin Institute of Technology, Harbin 150080, China; 3 Academy of Fundamental and Interdisciplinary Sciences, Harbin Institute of Technology, Harbin 150080, China

## Abstract

In this study, interactive effects of microstructure and cavity dimension on the filling behaviors in micro coining were investigated. The results indicate that the filling ability is dependent on both the cavity width *t* and the ratio of cavity width to grain size *t/d* strongly. The critical ratio *t/d* for the worst filling ability increases with cavity width *t* and tends to disappear when the cavity width *t* increases to 300 μm. A polycrystalline filling model considering the friction size effect, effect of constrained grains by the tools, grain size, cavity width and ratio of cavity width to grain size is proposed to reveal the filling size effect in micro coining. A quasi *in-situ* Electron Backscatter Diffraction (EBSD) method is proposed to investigate filling mechanism in micro coining. When several grains across the cavity width, each grain deforms heterogeneously to ordinate the deformation compatibility. When there is only one grain across the cavity width, the grain is fragmented into several smaller grains with certain prolongation along the extrusion direction to coordinate the deformation in the cavity. This is different from the understandings before. Then the filling deformation mechanism is revealed by a proposed model considering the plastic flow in micro coining.

Micro metal parts are widely applied in automotive, biomedical, consumer electronics and with the rapid development of micro electro-mechanical-systems (MEMS) and micro system technology (MST)^1^,^2^,^3^,^4^. In the last two decades, micro forming as a new micro manufacturing technology plays an important role on manufacturing micro metal parts. When the dimensions of metal parts down scale to micro scale, size effects occur and restrict the rapid development of micro forming^5^. It is needed to investigate the deformation behaviors in micro forming in-depth. Fu *et al.*^6^ found that the flow stress decreases and its scatter increases with the increase of grain size or decrease of specimen diameter by micro compression tests of pure cooper cylinders. The flow stress reduction with miniaturization can be interpreted by the surface models and modified surface models^1^,^7^,^8^,^9^,^10^,^11^ based on the softening effect of surface grain with free surfaces. Wang *et al.*^12^ revealed the flow stress scatter in micro compression through the proposed model considering orientation distribution of the surface grain. Wang *et al.*^13^,^14^ found that the flow stress increases when there are less than 3-4 grain across the specimen diameter in micro compression and revealed the mechanism through the proposed model considering the effect of surface, inner and constrained grains. Chan *et al.*^15^ found that the degree of inhomogeneous deformation increases with the increase of grain size in micro extrusion process of pure copper. Cao *et al.*^16^ found that the extruded micro pins curves when using the coarse grained materials. Lin *et al.*^17^ proposed a model based on crystal plasticity theory to reveal the curvature in micro extrusion of coarse grained materials. Meng *et al.*^18^ manufactured a multi-level flanged part is produced via progressive micro extrusion and blanking and investigated the effect of grain size on the microstructure evolution and fracture behaviors in progressive micro forming. Meng *et al.*^19^ also investigated the microstructure evolution of commercially pure titanium in thermal-aided meso forming of a dental abutment. The surface grains on the square extrudate generate an equiaxed structure because of severe deformation, reflecting that meso forming at elevated temperature facilitates the homogenization of material flow without coarsening grain size. Kim *et al.*^20^ manufactured micro gear shaft with good quality through ECAP process. Wang *et al.*^21^ developed a feature-based method for defect-free cold-forging process to manufacture a non axisymmetrical micro part. Yang *et al.*^22^ studied the effect of high-energy assistance on the micro deep drawing and micro forging processes. The formability and the surface roughness were improved. Wang *et al.*^23^ manufactured a micro turbine by isothermal micro forging process. A micro turbine with a higher micro blade is manufactured when using the circular ring preform compared to that using the circular one. To reveal the deformation mechanism in-depth, Wang *et al.*^24^ investigated the effect of the ratio of cavity width to grain size on the filling behavior through micro coining process. It indicated that the filling behavior is the worst when where are only about 2 grains across the cavity width at elevated temperature. Wang *et al.*^25^ found the similar results in micro coining at room temperature. The similar filling size effect was also found. Ast *et al.*^26^ investigated the microstructure evolution of three different grained materials in nano coining process by electron back scatter diffraction (EBSD). The results indicated that strong orientation gradients occurred below the cavities for single crystal, a sub-grain formation inside and around the cavities for the ultrafine grain (UFG) samples and only a slight elongation of the grains inside the cavity was found for the nanocrystalline material. Based on the above literature review, it is found that various researches have been explored and the focus is on the mechanical size effects and material plastic flow. During the micro forming process, the intergranular and intragranular deformation behaviors are remain unknown and need to be explored. In this study, micro coining tests of pure nickel were conducted to investigate the interactive effects of microstructure and cavity width on the filling behavior. The filling size effect occurs when there are only a few grains across the cavity width. A quasi *in-situ* EBSD method is proposed to investigate the filling behavior and reveal the filling mechanism by a model based on the crystalline plasticity deformation in micro coining.

## Results and Discussions

### Filling size effect

**Figure 1:**
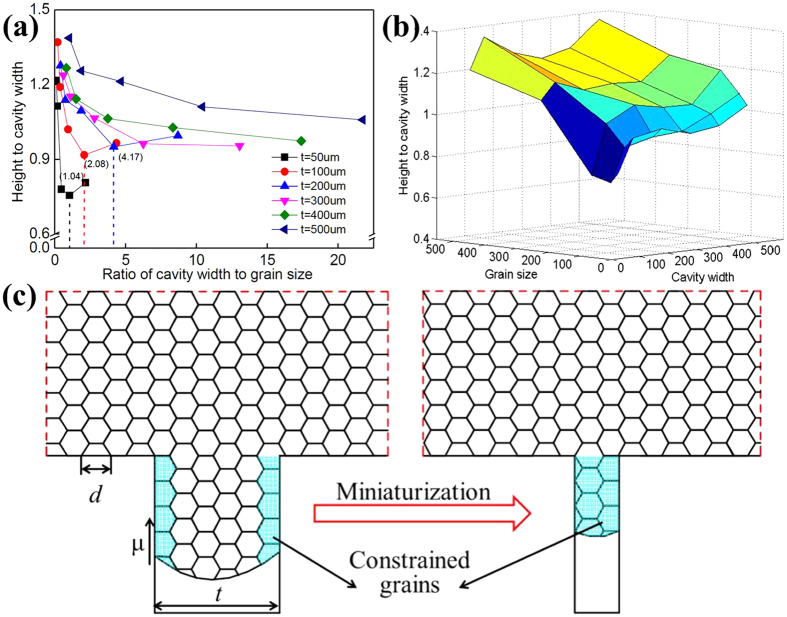
(**a**) Relationship between the ratio of height of micro rib to grain size and the ratio of height of micro rib to cavity width (**b**) effects of cavity widths and grain sizes on relative heights of micro ribs (**c**) polycrystalline model in micro coining with miniaturization.

Figure 1(a) shows the relationship between the ratio of cavity width to grain size and the ratio of height of micro rib to cavity width. It indicates that the ratio of height of micro rib to cavity width tends to decrease first then increase with the decrease of the ratio of cavity width to grain size when the cavity width in the range of 50–200 μm. The critical ratios of cavity width to grain size are 1.04, 2.08 and 4.17, respectively. The critical ratios are dependent on the cavity width. The worst filling property is not only related to the numbers of grains across the cavity, but also the cavity width. This is different from the findings in references^24^,^25^, which confirms that the critical ratio of cavity width to grain size for the worst filling property in micro coining is a constant. When the cavity width increases to 300, 400 and 500 μm, there is no critical ratio. The ratio of height of micro rib to cavity width just increases with the decrease of the ratio of cavity width to grain size monotonously. In this situation the ratios of specimen width to cavity width are 7, 8.75 and 11.7, respectively. The plastic deformation mode has changed from coining and extrusion.

The filling behaviors are the results of interaction of grain size, cavity width and ratio of cavity width to grain size, as shown in Fig. 1(b). The ratio of height to cavity width reaches the maximum when the cavity width and grain size are the maximal values in this study. The relative filling height is small when both the cavity width and grain size are small, which not only dependents on the cavity width, but also the grain size and the grain numbers across the cavity width. To analysis the filling size effect, a polycrystalline model in micro coining is built, as shown in Fig. 1(c). Several parameters influence the filling ability in micro coining. A specimen with smaller grain size results in higher strength and contributes to material filling negatively. A die with smaller cavity width and ratio cavity width to grain size lead to more difficult to fill the cavity because of the constraint from the tools^13^,^14^ and friction size effect^27^,^28^,^29^,^30^, respectively. Interactive effects of these parameters result in the final filling ability in micro coining. Thus, when both the cavity width and grain size are relative large, both the deformation resistance and the friction factor are relative low. Correspondingly, the relative filling height reaches to a maximum. On the opposite, the relative filling height reaches to a minimum, as shown in Fig. 1(a). The grain fraction constrained by the tools increase with the increase of grain size or decrease with the increase of cavity width. When there are less than 2 grains across the cavity width (Fig. 1(c)), all the grains are constrained by the tools, the constrained grain fraction reaches a maximum. The strengthening effect by the constrained grains and friction size effect result in the worst filling ability, as shown in Fig. 1(a) for cavity width of 50 and 100 μm. The difference of the critical ratios is resulted from the difference of friction factors between the cavity width of 50 and 100 μm in micro coining. When there is less one grain across the cavity width, the filling ability is even better. This occurrence of the phenomena is attributed to the difference of ability between intergranular and intragranular coordinated deformation. The ability of coordinated deformation inner grain is easier than that of among grains. Thus, a better filling ability is obtained in this region. And the physical mechanism about the phenomena will address in detail in the next section.

### Quasi *in-situ* EBSD analysis

**Figure 2:**
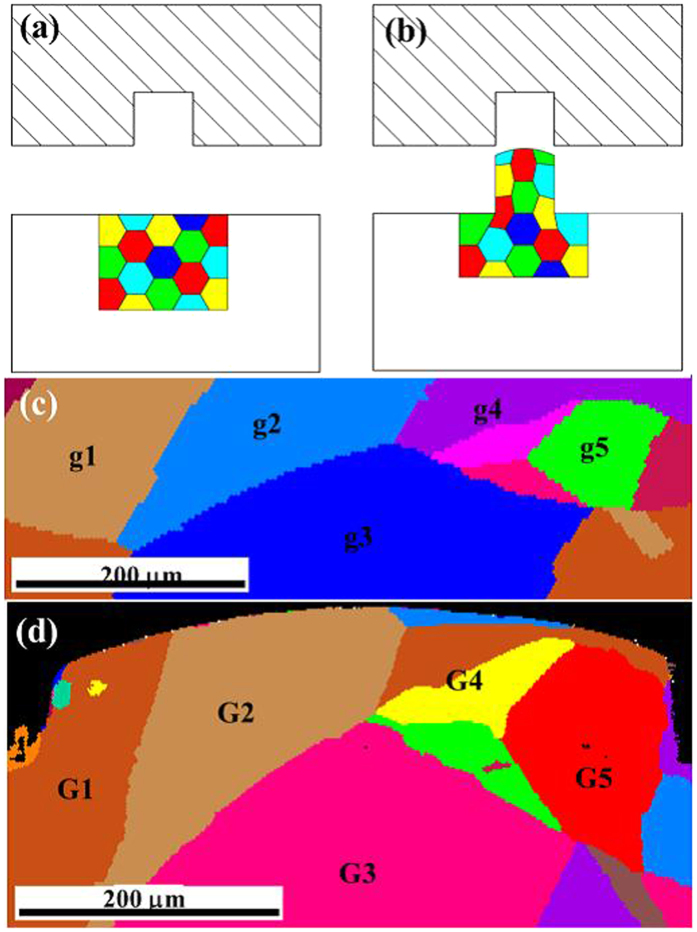
Schematic design of quasi *in-situ* EBSD studies of grain distributions before and after micro coining. (**a**) diagram of microstructure before deformation (**b**) diagram of microstructure after deformation. (**c**) microstructure by quasi *in-situ* EBSD before deformation (**d**) microstructure by quasi *in-situ* EBSD after deformation.

In micro coining, there are usually only several grains or even only one grain across the cavity width. The deformation behaviors dependent on the microstructure distribution are inhomogeneous because of heterogeneous materials in the deformation region. It is difficult to analyze the filling behavior using the traditional models. Thus, the *in-situ* or quasi *in-situ* observation and analysis are needed and necessary. To trace the deformation behaviors of individual grain in micro coining, a quasi *in-situ* EBSD method is proposed in micro forming, as shown in Fig. 2. The rectangular sample is placed in the experimental die. The main deformation of the sample is focused on the area approaching the die cavity. One side of the sample in the thickness direction is polished and an area approaching the die cavity on the surface is scanned using EBSD, as shown in Fig. 2(a). The scanned region is marked by a marker pen on the other side of the sample. Then the sample is placed in the dies and pressed by a punch at a load of 30 *k*N and velocity of 0.36 mm/min. The deformed sample is taken out from the dies and scanned using EBSD again on the same area above, as shown in Fig. 2(b). Thus, the distributions of grains in the tracing area before and after micro coining are obtained as shown in Fig. 2(c,d) under cavity width of 500 μm and grain size 490 μm (average confidence index 0.41 for micro coined part). In this study, 5 grains, denoted g1-g5 before deformation, were clearly identified, as marked out in Fig. 2(c). After deformation, the five grains are denoted G1-G5 as shown in Fig. 2(d). The numbers in the Fig. 2(c) of specimen before deformation and Fig. 2(d) of specimen after deformation are in one-to-one correspondence. Through the proposed quasi *in-situ* EBSD method, the evolution of individual grain in the deformation region is traced. The deformation behaviors of the individual grain can be analyzed by comparison the microstructural characteristics before and after deformation. From Fig. 2(c,d), the shapes of the grains approaching the cavity corners are changed after deformation, which indicate that these grains undergo obvious plastic deformation after deformation. The further analysis of the microstructure revolution and plastic deformation behavior will be addressed in the next part.

**Figure 3:**
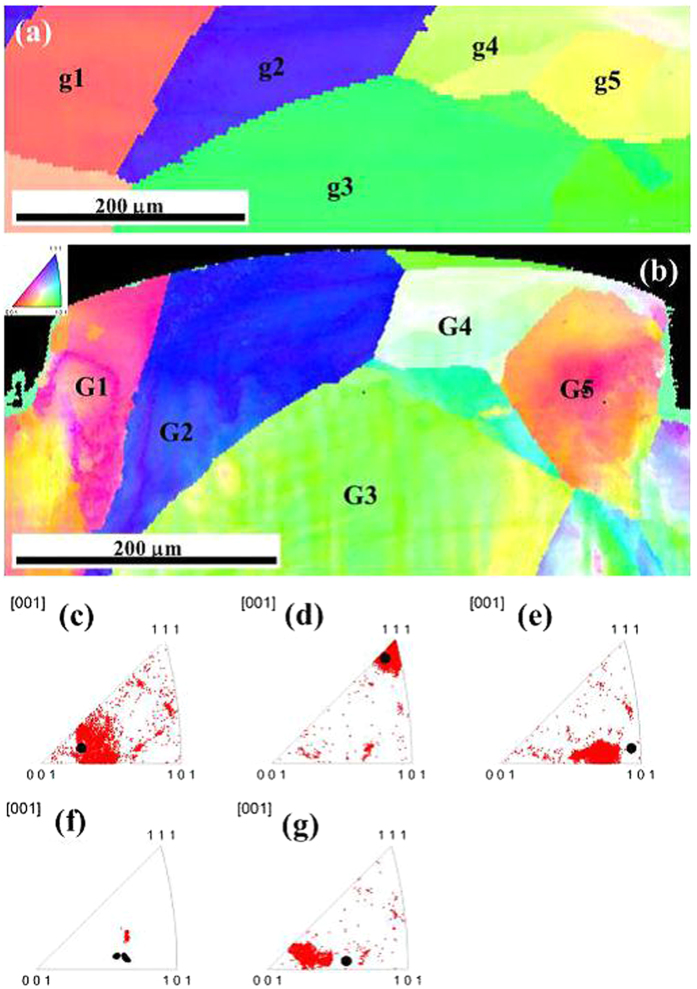
Evolution of grain orientations parallel to the loading direction of grains G1–G5 under deformation. (**a**) global grain orientation distributions before deformation (**b**) global grain orientation distributions after deformation. (**c**–**g**) are orientation of individual grain before and after deformation.

Figure 3 shows the grain orientation distribution maps in the deformation region of the specimen before and after micro coining. From Fig. 3(a,b), the inner grain orientation of each grain is uniform before deformation. The orientations of marked grains (G1-G5) were coded as disordered colors after deformation. The grain orientation of individual grain is changed with different degree after micro coining. The degree of changing is related to the location of individual grain. The orientation distributions of pixels in the loading direction within each of the 5 grains are expressed in inverse pole. The orientation distribution is visualized by color coding, as illustrated in the top left corner of Fig. 3(b), i.e., red for [100], green for [101] and blue for [111]. By following the individual grain, it is evident that orientations of the grains varied, indicating lattice rotation during the deformation process. Multiple sub-regions with different orientations also developed in some grains, e.g., grain G1 and grain G5 (Fig. 3(b)), indicating obvious heterogeneous plastic deformation within an individual grain. Fig. 3(c–g) shows the orientation distribution of pixels in the loading direction within each of the 5 grains, as expressed in inverse pole figures. The black spot in (c) to (g) is represented the original orientation of individual grain. By comparison, the orientation is dispersed in each grain after deformation, revealing that the plastic deformation is inhomogeneous even in the same grain. Grain G1 changes its orientation from approaching the <001>to the lines <101>-<001> and <111>-<001> in two directions. The upper part of grain G1 rotates to the orientation of line <001>-< 111>, the bottom part of grain G1 rotates to the orientation of line <001>-<101>. Grain G2 rotates from approaching the <111> to the <111> along the line <001>-<111>. Grain G3 rotates from approaching the <101> to the line <001>-<101>. Grain G4 rotates towards <111> globally. Grain G5 rotates from the line <001>-<101> to the line <001>-<111>. Misorientations in the inner grain occur after deformation and attribute to maintain the deformation compatibility by intergranular and intragranular heterogeneous deformation in this situation.

**Figure 4:**
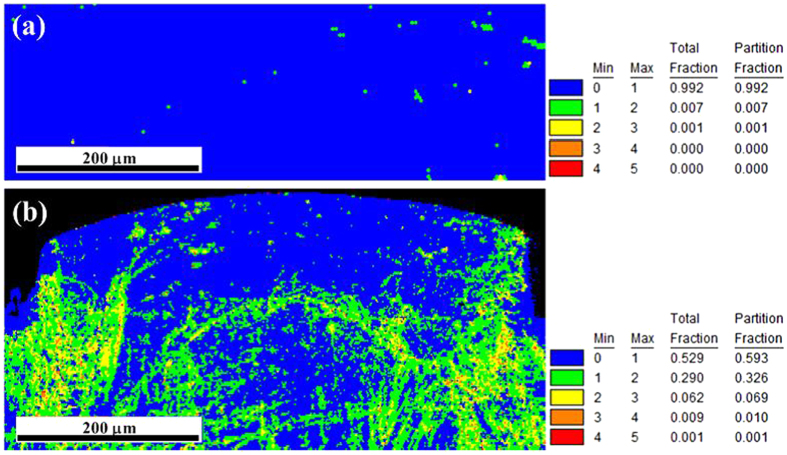
KAM values distributions. (**a**) before micro coining (**b**) after micro coining.

Kernel average misorientation (KAM) as characterization of local misorientation is calculated by averaging the misorientation among the point at the center of the kernel to its nearest neighbors. KAM is commonly used as a tool in OIM (orientation imaging microscopy) to assess the magnitude of the residual plastic strain in the grains of the deformed metals^31^,^32^. KAM is correlated with the local plastic strain and the densities of geometrically necessary dislocation^33^,^34^,^35^. Therefore, when the plastic deformation among sub-regions with in a grain is homogeneous, a low KAM value (angle) is obtained. On the other hand, large deformation inhomogeneity among the sub-region of a grain leads to a large KAM value. The neighboring points with an orientation difference of 5° or larger than the value measured from the center is excluded from the kernel. In general, KAM values are low (<\1°) in recrystallized grains and high (>1°) in deformed grains. Figure 4 shows the KAM values distributions maps of the same region shown in Fig. 3 before and after micro coining. From Fig. 4(a), it is clearly seen that the value of KAM of all the grains is very low (<1°) before deformation. It can be regarded as there is no plastic strain before deformation. This is in accordance with the testing material treated by heat treatment of complete recrystallization. After deformation, the distribution of values of KAM is inhomogeneous in different grains and different regions of the individual grain. The higher values (1–4°) of KAM are mainly distributed at the sites approaching the entrance angle of micro die and boundaries of inner grain, as shown in Fig. 4(b). It is also evident that within each grain, KAM values are varied, indicating inhomogeneity of the plastic deformation within individual grains. At the top of the micro rib, the values of KAM are near to zero. This means that there is no obvious plastic strain after deformation. Combining the analysis of grain orientation in Fig. 3(f), although the grains at the top of the micro rib are not deformed, but the orientation is changed after deformation to coordinate the inhomogeneous intergranular deformation. The high values of KAM at the grain boundaries after deformation mean that the inner grains are deformed firstly during deformation. From the analysis of the distribution of the KAM values, the deformation is inhomogeneous in different grains, different regions of the individual grain and grain boundaries.

**Figure 5:**
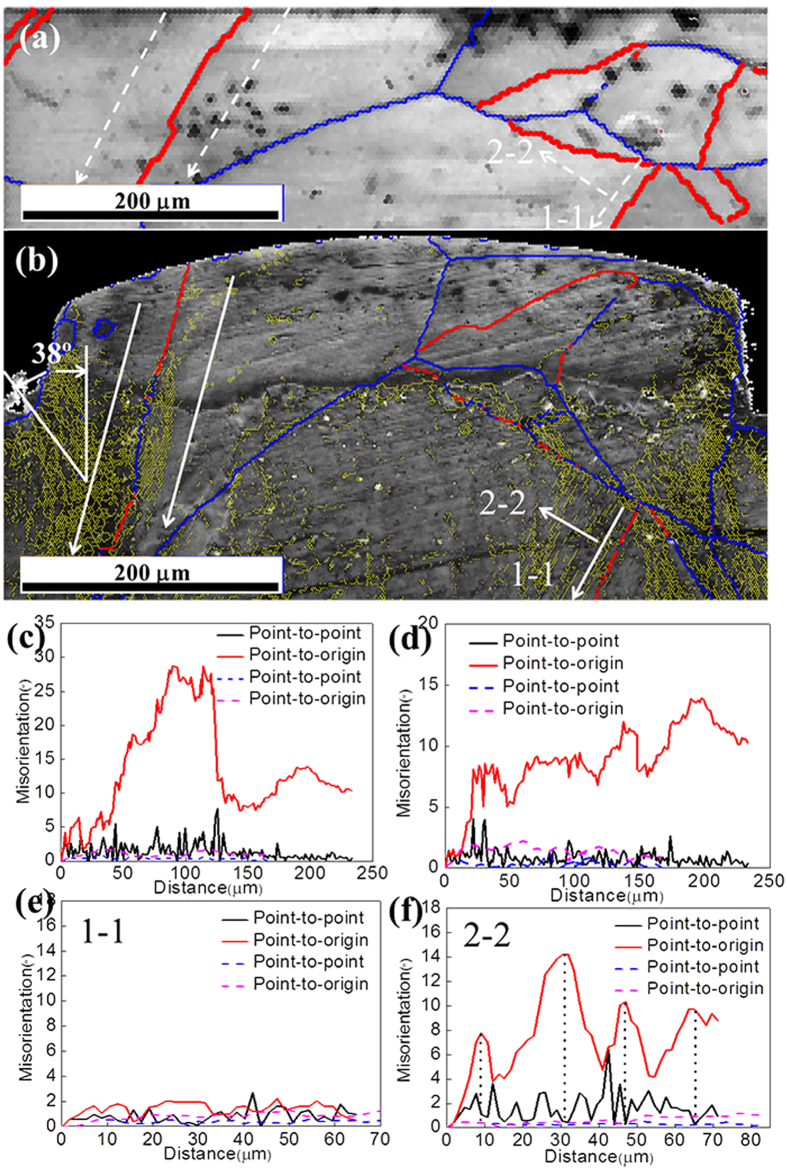
Grain boundary distributions before (**a**) and after (**b**) micro coining. The notches (**c,d**) are the global grain orientation distributions before and after deformation. The notches (**e,f**) are orientation of individual grain before and after deformation. The black spot in (**c–f**) is represented the original orientation of individual grain. The dash lines represent the misorientation after deformation, the solid lines represent the misorientation before deformation.

Figure 5 shows the grain boundary distributions before and after micro coining. It indicates that a lot of small angular grain boundaries are formed after micro coining approaching the entrance corner of micro die and inner grain boundaries. The twin boundaries approaching the entrance corner of micro die change to general large angle boundaries, which resulted from the severe deformation in the region. Figure 5(c,d) show the misorientations of grains G1 and G2 in the longitudinal direction. Point-to-point and point-to-origin misorientation measurements in a line trace across a single grain. The misorientations of point-to-origin decreases sharply from about 30° to 10° at the corner with an angle of 38° compared to the extrusion direction. These changes indicate that there is an evidence shear band because of severe plastic deformation in the entrance corner of the micro cavity. For grain G3, at the side of the twin grain boundary, the point-to-origin and point-to-point after deformation are not changed compared to those before deformation parallel to the grain boundary. The point-to-point after deformation is changed slightly compared to that before deformation perpendicular to the grain boundary. The point-to-origin after deformation is changed periodicity compared to those before deformation perpendicular to the grain boundary. In Fig. 5(b), it also can be seen that many small angular boundaries (yellow lines) are distributed parallel to the grain boundary. These results indicated the deformation of inner grain G3 is slight. Approaching to the region of the grain boundary, the single slip is the main deformation model.

Based on the analysis above, when there are several grains across the cavity width, the filling behavior is complicated because of the heterogeneous microstructure coupling the effect of processing parameters. The deformation degree is dependent on the grain distribution strongly. The constrained grains by the tools deformed heavily, the grains with free surface just changing their orientations to coordinate the inhomogeneous intergranular deformation with slightly plastic deformation. Grains in the inner part across the cavity width, the role the grains is to coordinate the inhomogeneous intergranular deformation by rotation and slip with their neighbouring grains. Therefore, the filling behavior is accomplished through intergranular and intragranular heterogeneous deformation to maintain the deformation compatibility and fill the cavity when there are several grains across the cavity width.

**Figure 6:**
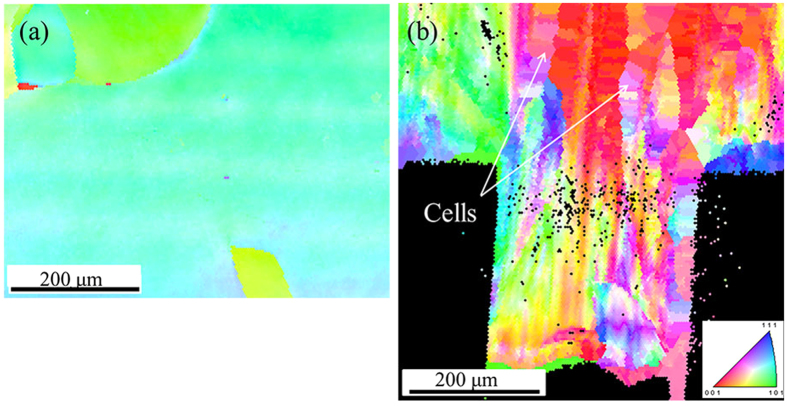
Grain orientation distributions. (**a**) before micro coining (**b**) after micro coining.

Figure 6 shows grain orientation distributions of only one grain across the cavity width before and after micro coining (average confidence index 0.22 for micro coined part). From Fig. 6(a), the orientation of the biggest grain in the region is uniform before deformation. The orientation distributions of pixels in the loading direction of the grain are expressed in the inverse pole. The orientation distribution is visualized by color coding, as illustrated at the bottom left corner of Fig. 6(a), i.e., red for [100], green for [101] and blue for [111]. By following the grain, it is evident that orientations of the grain rotated from original <562> to <001> after deformation. The <001> orientation is consistence with the material flowing direction. That means the crystal orientations of the grains in the deformation region tend to rotate to the material flowing direction. It is clearly also indicated that there are many zones and cells formed after deformation (as shown in Fig. 6(b)). The zones with longitudinal distribution are formed and many cells with transverse distribution are formed in the zones after deformation. The formed longitudinal zones and transverse cells are related to the plastic flow during the deformation process.

**Figure 7:**
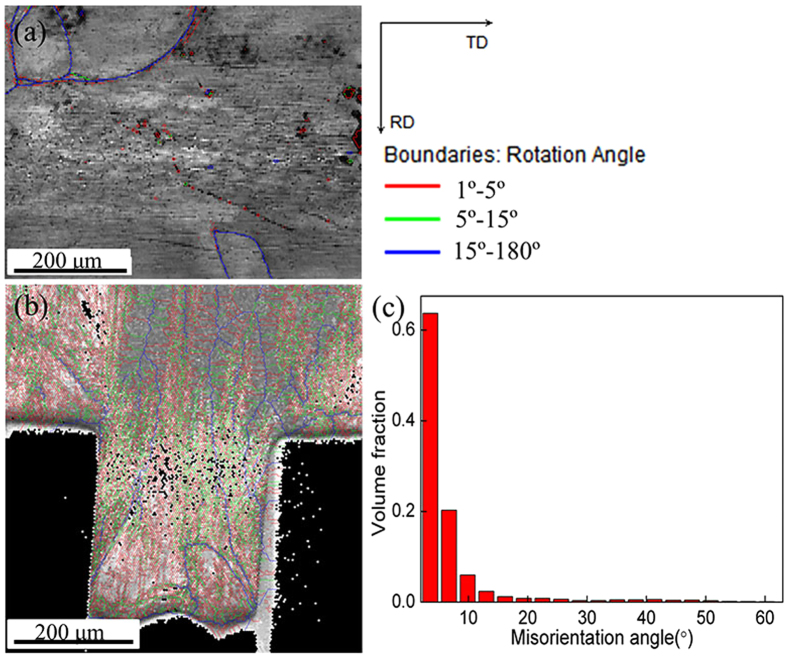
Grain boundary distributions. (**a**) before micro coining (**b**) after micro coining (**c**) misorientation angle distributions after micro coining.

Figure 7 shows the grain boundary distributions before and after micro coining. It indicates that a lot of small and large angular grain boundaries are formed after micro coining. The large angular grain boundaries (LAGBs) are mainly distributed along the extrusion direction and formed new smaller grains. The small angular grain boundaries (SAGBs) are mainly distributed perpendicular to the extrusion direction. The new formed grains are divided into small sub-grains by the small angular grain boundaries (as shown in Fig. 6(b)).

**Figure 8:**
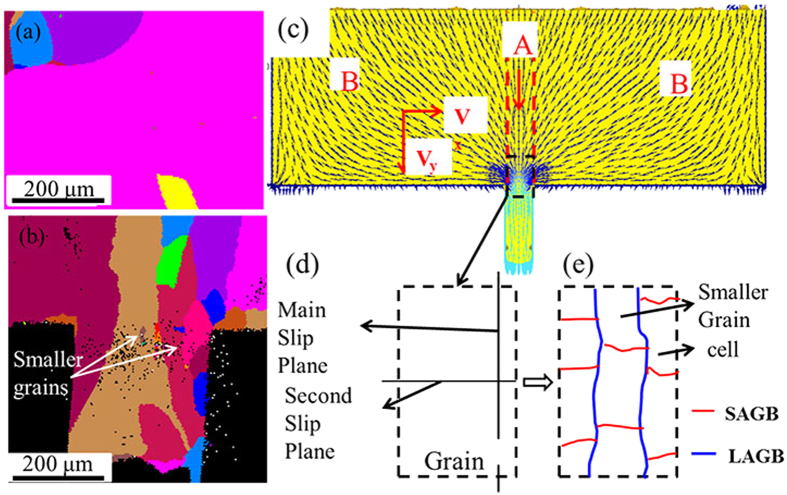
(**a**) Grain distribution before micro coining (**b**) grain distribution after micro coining (**c**) velocity field distribution during the micro coining process (**d**) main and second slip planes (**e**) large and small angular grain boundaries during deformation.

Figure 8(a,b) show the grain distributions before and after micro coining process when there is only one grain across the cavity width in micro coining. It is clearly indicated that there is only one big grain across the cavity width before deformation. The main deformation is mainly focused on the interior of the big grain. The effect of the neighboring grains on the big grain is limited and can be neglected because of that the neighboring grains are far from the big grain. After deformation, as shown in Fig. 8(b), the origin big grain is fragmented into several smaller grains. The smaller grains are elongated along the direction of material flowing into the groove. The mechanism of grain fragmentation and sub-grains formation are mainly dependent on the plastic flow pattern inducing shearing deformation. Figure 8(c) shows the velocity distribution of plastic flow in micro coining process. The flowing direction at the region A is parallel to the extrusion direction of the micro rib. The flow velocity distribution characteristic from the center to the edge across the cavity width is lamellar. The largest flow velocity is in the center of the micro rib and the flowing velocity decreases from the cavity center to the wall because of friction from the cavity walls. The material in the deformation region A can be separated into several thin layers according to the lamellar distribution of the flow velocity. Shearing deformation occurs at the interfaces of the interlaminar materials during deformation. The flowing direction at the region B is inclined to the extrusion direction of the micro rib. The flowing velocity at the region B can be separated into two parts: (1) along the extrusion direction (*V*_x_) and (2) parallel to the extrusion direction (*V*_y_). The flowing velocity *V*_x_ at the region B will induce the transverse flow of the materials in the region A. The main and second slip planes are corresponding to the longitudinal and transverse directions, as shown in Fig. 8(d). The shearing deformation will result in the formation of the large and small angular grain boundaries during deformation, as shown in Fig. 7(b). Then new grains are formed along the extrusion direction (Fig. 8(b)) and the cells are formed in the smaller grains (Fig. 6(b)).

## Conclusions

In this study, the interactive effects of microstructure and cavity width on the filling ability of pure nickel polycrystals in micro coining were investigated. The conclusions are drawn as follows:

1. The filling size effect is dependent on both the cavity width *t* and the ratio of cavity width to grain size *t*/*d* strongly. The coupling effects of microstructure and cavity dimension results in the worst filling ability when *t* is 50 μm and *t/d* is 1.04. The critical ratio *t*/*d* increases with the cavity width *t* and tends to disappear when the cavity width *t* increases to 300 μm, because of the transformation of the deformation patterns from micro coining to micro extrusion.
2. A polycrystalline filling model in micro coining is proposed considering the friction size effect, the effect of constrained grains by the tools and grain size, cavity width, ratio of cavity width to grain size. These parameters contribute negative or positive effect individually on the filling ability and their competition leads to the filling size effect in micro coining.
3. A quasi *in-situ* EBSD method is proposed to investigate the filling process in micro coining. The intergranular and intragranular deform heterogeneously to ordinate the compatibility of different grains in the cavity when several grains across the cavity width. However, smaller grains with certain prolongation distribute along the extrusion direction are formed when there is only one grain across the cavity width because of severe plastic deformation in micro coining. This finding is different from the previous reports and interpreted by a proposed model considering the severe shearing deformation in the cavity.

## Experimental

In this research, a female die with U-shape micro groove is designed to conduct the micro coining process and to investigate the deformation behaviors in micro coining. The dimensions of the micro grooves are 50, 100, 200, 300, 400 and 500 μm in width and with a specific ratio of groove depth to width of 3. Pure nickel with purity of 99.8% is selected to conduct the micro coining tests. The specimens were manufactured by a precision machining process. The dimensions of the rectangular specimen are 5.5 × 3.5 × 2.0 mm with a corner of R0.5. To homogenize the microstructure of the as-received materials, the material was treated at temperature of 500, 650, 900, 1000, 1025 and 1100 °C for 3 h, and then cooled in air. The grain sizes ranges from 13, 23, 48, 107, 267 and 490 μm, respectively. The micro coining tests were carried out by a Zwick testing machine with a load cell of 100 *k*N. To reduce the effect of friction on the deformation behaviors, all the tests were carried out with lubricant of castor oil. The specimens were pressed with a load of 10 to 40 *k*N and a low punch velocity of 0.36 mm/min was used for all tests in this research.

## Additional Information

**How to cite this article**: Wang, C. *et al.* Interactive effect of microstructure and cavity dimension on filling behavior in micro coining of pure nickel. *Sci. Rep.* **6**, 23895; doi: 10.1038/srep23895 (2016).
